# Supplementary material for: Effect of dietary concentrate to forage ratio on growth performance, rumen fermentation and bacterial diversity of Tibetan sheep under barn feeding on the Qinghai-Tibetan plateau
Source: PeerJ. 2019 Aug 5;7:e7462. doi: 10.7717/peerj.7462 (PMC6686838; doi:10.7717/peerj.7462)
Supplement: Supplemental Information 2 [file peerj-07-7462-s002.doc]

**Supplementary materials**

**Effect of dietary concentrate to forage ratio on rumen fermentation, bacterial diversity and growth performance of Tibetan sheep under barn feeding on Qinghai-Tibetan plateau**

Hongjin Liu^1,2,3^ ,Tianwei Xu^1,2^,, Li Ma^1,2,3^, Xueping Han^1,2,4^, Xungang Wang^1,2,3^, Xiaoling Zhang^1,2,3^, Linyong Hu^1,2^ , NaZhao^1,2^, Yongwei Chen^4^, Li Pi^1^, Xinquan Zhao^1,2^, Shixiao Xu^1,2^

^1^Northwest Institute of Plateau Biology, Chinese Academy of Sciences, Xining, 810008, China

^2^Key Laboratory of Adaptation and Evolution of Plateau Biota, Chinese Academy of Sciences, Xining, 810008, China

^3^University of Chinese Academy of Science, Beijing, 10049, China

^4^Technology Extension Service of Animal Husbandry of Qinghai, Xining, 81008, China

***Corresponding author:** prof. Shixiao Xu, Northwest Plateau Institute of Biology, Chinese Academy of science, 810001 Xining, China; E-mail:sxxu@nwipb.cas.cn

**Table S1** The nutrition of concentrate and forage feed in the experiment

| Items | Concentrate feed | Forage feed |
| --- | --- | --- |
| Ingredient (%) | |  |
| CP | 15.78 | 6.31 |
| EE | 3.92 | 2.13 |
| Starch(mg/g) | 43.42 | 25.58 |
| ADF | 5.11 | 34.1 |
| NDF | 13.89 | 57.64 |
| Calcium | 0.82 | 0.35 |
| Magnesium | 0.48 | 0.24 |
| Phosphorus | 0.57 | 0.22 |

CP=crude protein; EE= ether extract; ADF=acid detergent; NDF= natural detergent fiber.

**Table S2** The statistics of raw rate across the 20 different samples

| Sample Name | Raw PE | Combined | Qualified | Taxon Tags | OTUs | Q30 | GC% | Effective% |
| --- | --- | --- | --- | --- | --- | --- | --- | --- |
| HS1.1 | 83,751 | 81,974 | 80,575 | 77,412 | 1,836 | 98.40 | 53.26 | 95.81 |
| HS1.2 | 71,167 | 69,347 | 68,147 | 64,413 | 2,986 | 98.43 | 53.34 | 95.18 |
| HS1.3 | 84,189 | 82,243 | 80,774 | 75,358 | 2,876 | 98.26 | 53.31 | 95.36 |
| HS1.4 | 80,635 | 78,727 | 77,427 | 73,322 | 2,957 | 98.41 | 53.88 | 95.43 |
| HS2.1 | 68,348 | 66,703 | 65,502 | 62,400 | 2,559 | 98.37 | 53.16 | 95.34 |
| HS2.2 | 84,011 | 82,072 | 80,659 | 76,112 | 3,246 | 98.36 | 53.35 | 95.35 |
| HS2.3 | 78,049 | 76,242 | 75,028 | 71,101 | 2,836 | 98.48 | 53.45 | 95.71 |
| HS2.4 | 70,210 | 68,289 | 67,159 | 63,948 | 3,039 | 98.42 | 53.26 | 94.90 |
| HS3.1 | 71,873 | 70,097 | 68,868 | 65,248 | 3,022 | 98.29 | 53.39 | 95.13 |
| HS3.2 | 68,958 | 67,162 | 65,970 | 61,754 | 3,074 | 98.31 | 53.42 | 95.04 |
| HS3.3 | 70,010 | 68,356 | 67,247 | 63,251 | 2,860 | 98.36 | 53.53 | 95.3 |
| HS3.4 | 82,672 | 80,373 | 78,975 | 75,355 | 3,059 | 98.37 | 53.39 | 94.88 |
| HS4.1 | 69,154 | 67,489 | 66,445 | 63,817 | 2,611 | 98.40 | 53.19 | 95.64 |
| HS4.2 | 73,388 | 71,859 | 70,699 | 67,462 | 1,839 | 98.34 | 53.39 | 96.00 |
| HS4.3 | 67,758 | 66,318 | 65,063 | 62,529 | 1,753 | 98.31 | 52.73 | 95.73 |
| HS4.4 | 69,781 | 68,422 | 67,195 | 63,586 | 1,601 | 98.30 | 53.05 | 96.08 |
| HS5.1 | 69,879 | 68,441 | 67,334 | 64,434 | 1,728 | 98.41 | 53.64 | 96.03 |
| HS5.2 | 78,138 | 75,165 | 73,668 | 70,164 | 1,635 | 98.21 | 53.28 | 94.02 |
| HS5.3 | 74,180 | 72,582 | 71,389 | 68,478 | 1,967 | 98.36 | 52.97 | 95.93 |
| HS5.4 | 81,456 | 79,812 | 78,439 | 74,861 | 1,732 | 98.31 | 53.05 | 96.05 |

**Table S3** The relative abundance of the total phyla detected by taxonomic analysis (%)

| Taxonomy | Groups | | | | | avergege |
| --- | --- | --- | --- | --- | --- | --- |
|  | HS1 | HS2 | HS3 | HS4 | HS5 |  |
| Bacteroidetes | 50.0842 | 42.5576 | 45.3258 | 62.5178 | 60.4256 | 52.1822 |
| Proteobacteria | 22.2601 | 27.0355 | 23.7143 | 13.6692 | 15.0205 | 20.33992 |
| Firmicutes | 13.2894 | 13.7708 | 15.8411 | 15.884 | 12.915 | 14.34006 |
| Lentisphaerae | 6.1709 | 6.0401 | 4.9134 | 3.2629 | 6.6955 | 5.41656 |
| Fibrobacteres | 1.3113 | 1.8703 | 0.9493 | 0.7935 | 1.3266 | 1.2502 |
| Cyanobacteria | 0.9396 | 0.9117 | 1.1461 | 0.7502 | 1.4327 | 1.03606 |
| Acidobacteria | 1.344 | 1.6137 | 1.6489 | 0.2283 | 0.0049 | 0.96796 |
| Tenericutes | 1.3428 | 1.6294 | 1.6586 | 1.4655 | 1.3606 | 1.49138 |
| Actinobacteria | 0.872 | 1.5181 | 2.0857 | 0.3222 | 0.0227 | 0.96414 |
| Verrucomicrobia | 0.5093 | 0.4676 | 0.3048 | 0.0765 | 0.017 | 0.27504 |
| Spirochaetes | 0.4255 | 0.6789 | 0.4643 | 0.3729 | 0.4044 | 0.4692 |
| Gemmatimonadetes | 0.2708 | 0.4097 | 0.4571 | 0.0773 | 0.0028 | 0.24354 |
| Chloroflexi | 0.2672 | 0.368 | 0.4457 | 0.0769 | 0.0231 | 0.23618 |
| Nitrospirae | 0.2182 | 0.2725 | 0.1943 | 0.0356 | 0.002 | 0.14452 |
| Planctomycetes | 0.0919 | 0.1194 | 0.1838 | 0.0243 | 0.0057 | 0.08502 |
| Synergistetes | 0.0806 | 0.068 | 0.0587 | 0.066 | 0.034 | 0.06146 |
| Elusimicrobia | 0.0826 | 0.0992 | 0.0891 | 0.0356 | 0.0526 | 0.07182 |
| SHA-109 | 0.0065 | 0.0117 | 0.0113 | 0.0255 | 0.0045 | 0.0119 |
| Saccharibacteria | 0.0113 | 0.0389 | 0.0146 | 0.0194 | 0.0117 | 0.01918 |
| Chlamydiae | 0.0328 | 0.032 | 0.0267 | 0.0227 | 0.0336 | 0.02956 |
| Euryarchaeota | 0.0219 | 0.0194 | 0.0182 | 0.0186 | 0.0239 | 0.0204 |
| Latescibacteria | 0.032 | 0.0316 | 0.0267 | 0.0069 | 0 | 0.01944 |
| Chlorobi | 0.019 | 0.0385 | 0.03 | 0.0061 | 0 | 0.01872 |
| Candidate_division_SR1 | 0.0024 | 0.0202 | 0.0146 | 0.0085 | 0.0069 | 0.01052 |
| Armatimonadetes | 0.0109 | 0.0194 | 0.0166 | 0.002 | 0 | 0.00978 |
| Fusobacteria | 0.0049 | 0.0032 | 0.0138 | 0.0024 | 0.0061 | 0.00608 |
| Thermomicrobia | 0.0012 | 0.0073 | 0.0158 | 0.0016 | 0 | 0.00518 |
| JL-ETNP-Z39 | 0 | 0.0024 | 0.0109 | 0.0004 | 0 | 0.00274 |
| Thaumarchaeota | 0 | 0.0028 | 0.0053 | 0.0032 | 0.0008 | 0.00242 |
| Caldiserica | 0.0024 | 0.0065 | 0.0057 | 0 | 0 | 0.00292 |
| Parcubacteria | 0.0016 | 0.0024 | 0.0016 | 0 | 0 | 0.00112 |
| TM6 | 0.0024 | 0.0016 | 0.0004 | 0.0024 | 0 | 0.00136 |
| Candidate_division_OP3 | 0.002 | 0.0024 | 0.0008 | 0 | 0 | 0.00104 |
| WCHB1-60 | 0.0016 | 0.002 | 0.0012 | 0 | 0 | 0.00096 |
| Omnitrophica | 0.0008 | 0.0012 | 0 | 0 | 0 | 0.0004 |
| Hydrogenedentes | 0 | 0.0008 | 0 | 0 | 0 | 0.00016 |
| Deferribacteres | 0.0004 | 0.0004 | 0.0012 | 0 | 0 | 0.0004 |
| Aerophobetes | 0 | 0.0004 | 0.0004 | 0.0004 | 0 | 0.00024 |
| Thermotogae | 0.0004 | 0 | 0.0004 | 0 | 0 | 0.00016 |
| GOUTA4 | 0.0008 | 0 | 0 | 0 | 0 | 0.00016 |

The treatments HS1, HS2, HS3, HS4 and HS5 refer to the C:F ratios of 0:100, 15:85, 30:70, 45:55 and 60:40, respectively.

**Table S4** The relative abundance of total genus detected by taxonomic analysis (%)

| Taxonomy | Groups | | | | | Average |
| --- | --- | --- | --- | --- | --- | --- |
|  | HS1 | HS2 | HS3 | HS4 | HS5 |  |
| Prevotella_1 | 19.6225 | 18.3108 | 21.8010 | 39.1672 | 35.1513 | 26.8106 |
| Ruminobacter | 7.5876 | 6.0887 | 7.6767 | 3.4465 | 5.3562 | 6.0311 |
| Succinivibrionaceae_UCG-002 | 5.2903 | 11.0201 | 6.0547 | 6.1211 | 7.0781 | 7.1128 |
| Rikenellaceae_RC9_gut_group | 6.2063 | 4.6847 | 5.4145 | 3.5053 | 3.8518 | 4.7325 |
| Succinivibrio | 0.8221 | 1.6895 | 0.8178 | 0.3633 | 1.1071 | 0.9599 |
| Erysipelotrichaceae_UCG-004 | 1.5265 | 1.1530 | 1.2069 | 0.8798 | 0.6796 | 1.0892 |
| Prevotellaceae_UCG-003 | 2.6476 | 1.9713 | 2.1872 | 3.2122 | 3.2230 | 2.6483 |
| Fibrobacter | 0.9846 | 1.7375 | 0.8906 | 0.5608 | 0.8669 | 1.0081 |
| Ruminococcus_2 | 0.5387 | 0.3816 | 0.5921 | 1.2561 | 0.4129 | 0.6363 |
| Prevotellaceae_UCG-001 | 0.9592 | 0.7589 | 1.0963 | 1.7025 | 1.6317 | 1.2297 |
| Christensenellaceae_R-7_group | 1.1810 | 0.9565 | 1.5967 | 1.3359 | 1.0245 | 1.2189 |
| Ruminococcaceae_NK4A214_group | 1.0499 | 0.8788 | 1.4887 | 1.2409 | 0.8037 | 1.0924 |
| Succinimonas | 0.1738 | 0.3282 | 0.8048 | 0.0675 | 0.2348 | 0.3218 |
| Selenomonas_1 | 0.2737 | 0.2580 | 0.3643 | 1.0504 | 0.2348 | 0.4362 |
| Ruminococcaceae_UCG-014 | 0.6866 | 0.5565 | 0.7875 | 0.8588 | 0.4669 | 0.6713 |
| Saccharofermentans | 0.5484 | 0.4092 | 0.7935 | 0.6526 | 0.3492 | 0.5506 |
| Prevotellaceae_NK3B31_group | 0.1328 | 0.0680 | 0.0707 | 0.2035 | 0.4059 | 0.1762 |
| Victivallis | 0.7044 | 0.7892 | 0.6251 | 0.4118 | 0.3897 | 0.5840 |
| Ruminococcaceae_UCG-010 | 0.7233 | 0.7773 | 0.7875 | 0.5209 | 0.5997 | 0.6817 |
| Succiniclasticum | 0.2936 | 0.1895 | 0.3352 | 0.4016 | 0.2942 | 0.3028 |
| Butyrivibrio_2 | 0.2478 | 0.1927 | 0.3643 | 0.2845 | 0.2958 | 0.2770 |
| [Eubacterium]_coprostanoligenes_group | 0.3876 | 0.3924 | 0.4334 | 0.3703 | 0.2785 | 0.3724 |
| Treponema_2 | 0.2963 | 0.4561 | 0.3147 | 0.2170 | 0.2251 | 0.3018 |
| Lachnospiraceae_XPB1014_group | 0.3034 | 0.1846 | 0.3897 | 0.2774 | 0.2057 | 0.2722 |
| Quinella | 0.1598 | 0.1063 | 0.1382 | 0.2553 | 0.0907 | 0.1501 |
| SP3-e08 | 0.3206 | 0.2769 | 0.2003 | 0.1263 | 0.1727 | 0.2194 |
| Ruminococcaceae_UCG-002 | 0.3449 | 0.4043 | 0.3217 | 0.3174 | 0.1959 | 0.3168 |
| Ruminococcus_1 | 0.3260 | 0.2747 | 0.3973 | 0.2888 | 0.2359 | 0.3045 |
| Ruminococcaceae_UCG-013 | 0.2262 | 0.1970 | 0.2828 | 0.1393 | 0.1706 | 0.2032 |
| Lachnospiraceae_ND3007_group | 0.1851 | 0.1220 | 0.1932 | 0.1117 | 0.1166 | 0.1457 |
| Lachnospiraceae_AC2044_group | 0.1797 | 0.1447 | 0.2656 | 0.1592 | 0.1506 | 0.1800 |
| Thalassospira | 0.2753 | 0.3298 | 0.2780 | 0.1101 | 0.1252 | 0.2237 |
| Ruminiclostridium_5 | 0.1884 | 0.1544 | 0.2256 | 0.1436 | 0.1312 | 0.1686 |
| Prevotellaceae_YAB2003_group | 0.1090 | 0.1317 | 0.1090 | 0.2218 | 0.1657 | 0.1475 |
| Ruminococcaceae_UCG-005 | 0.2051 | 0.1420 | 0.2305 | 0.1506 | 0.1808 | 0.1818 |
| Prevotellaceae_UCG-004 | 0.1004 | 0.0853 | 0.1441 | 0.1058 | 0.1150 | 0.1101 |
| Pseudobutyrivibrio | 0.1716 | 0.1576 | 0.2321 | 0.1484 | 0.1479 | 0.1715 |
| Anaeroplasma | 0.1959 | 0.1927 | 0.2478 | 0.2024 | 0.1490 | 0.1976 |
| Pseudomonas | 0.0335 | 0.1220 | 0.0686 | 0.0049 | 0.0065 | 0.0471 |
| Prevotellaceae_Ga6A1_group | 0.1285 | 0.0874 | 0.0772 | 0.2332 | 0.1301 | 0.1313 |
| Sphingomonas | 0.0874 | 0.1797 | 0.1501 | 0.0000 | 0.0005 | 0.0836 |
| [Eubacterium]_ruminantium_group | 0.1652 | 0.0837 | 0.2111 | 0.1382 | 0.1144 | 0.1425 |
| Lachnospiraceae_NK3A20_group | 0.1301 | 0.1258 | 0.1517 | 0.1236 | 0.1641 | 0.1390 |
| Sphaerochaeta | 0.0529 | 0.0853 | 0.0410 | 0.0394 | 0.0286 | 0.0494 |
| Papillibacter | 0.1285 | 0.0939 | 0.1398 | 0.1026 | 0.1090 | 0.1148 |
| Lachnospiraceae_NK4A136_group | 0.0448 | 0.0189 | 0.0356 | 0.0766 | 0.0200 | 0.0392 |
| Fretibacterium | 0.0869 | 0.0675 | 0.0578 | 0.0556 | 0.0308 | 0.0597 |
| Desulfovibrio | 0.0766 | 0.0739 | 0.0637 | 0.0799 | 0.0464 | 0.0681 |
| Anaerosporobacter | 0.0378 | 0.0356 | 0.0448 | 0.0151 | 0.0993 | 0.0465 |
| Acetitomaculum | 0.0950 | 0.0513 | 0.1198 | 0.0718 | 0.0605 | 0.0797 |
| probable_genus_10 | 0.0615 | 0.0524 | 0.0891 | 0.0572 | 0.0696 | 0.0660 |
| Anaerovorax | 0.0642 | 0.0707 | 0.0793 | 0.0524 | 0.0540 | 0.0641 |
| Acidibacter | 0.0626 | 0.0464 | 0.0729 | 0.0011 | 0.0011 | 0.0368 |
| Anaerovibrio | 0.0437 | 0.0659 | 0.0777 | 0.0605 | 0.0421 | 0.0580 |
| Candidatus_Saccharimonas | 0.0146 | 0.0184 | 0.0157 | 0.0173 | 0.0097 | 0.0151 |
| Elusimicrobium | 0.0524 | 0.0659 | 0.0723 | 0.0238 | 0.0302 | 0.0489 |
| Moryella | 0.0561 | 0.0356 | 0.0513 | 0.0491 | 0.0405 | 0.0465 |
| Ruminococcaceae_UCG-001 | 0.0211 | 0.0167 | 0.0173 | 0.0119 | 0.0389 | 0.0212 |
| Oscillibacter | 0.0016 | 0.0027 | 0.0043 | 0.0259 | 0.0005 | 0.0070 |
| Veillonellaceae_UCG-001 | 0.0281 | 0.0200 | 0.0372 | 0.0351 | 0.0189 | 0.0279 |
| U29-B03 | 0.0313 | 0.0318 | 0.0399 | 0.0448 | 0.0313 | 0.0358 |
| Candidatus_Hepatincola | 0.0545 | 0.0432 | 0.0383 | 0.0146 | 0.0184 | 0.0338 |
| Spirochaeta_2 | 0.0486 | 0.0378 | 0.0378 | 0.0146 | 0.0308 | 0.0339 |
| unidentified_Gastranaerophilales | 0.0178 | 0.0167 | 0.0092 | 0.0065 | 0.0275 | 0.0155 |
| Bibersteinia | 0.0248 | 0.0124 | 0.0146 | 0.0043 | 0.0059 | 0.0124 |
| Lachnoclostridium_10 | 0.0389 | 0.0480 | 0.0318 | 0.0189 | 0.0162 | 0.0308 |
| Alloprevotella | 0.0227 | 0.0130 | 0.0200 | 0.0324 | 0.0173 | 0.0211 |
| Ruminiclostridium_9 | 0.0103 | 0.0027 | 0.0173 | 0.0167 | 0.0038 | 0.0101 |
| Pantoea | 0.0313 | 0.0356 | 0.0205 | 0.0097 | 0.0157 | 0.0226 |
| Acinetobacter | 0.0070 | 0.0135 | 0.0086 | 0.0016 | 0.0016 | 0.0065 |
| Lactobacillus | 0.0092 | 0.0119 | 0.0043 | 0.0173 | 0.0005 | 0.0086 |
| Candidatus_Methanomethylophilus | 0.0124 | 0.0130 | 0.0070 | 0.0076 | 0.0065 | 0.0093 |
| Alistipes | 0.0038 | 0.0022 | 0.0022 | 0.0130 | 0.0022 | 0.0046 |
| Oscillospira | 0.0270 | 0.0194 | 0.0232 | 0.0135 | 0.0130 | 0.0192 |
| [Anaerorhabdus]_furcosa_group | 0.0140 | 0.0108 | 0.0205 | 0.0162 | 0.0124 | 0.0148 |
| Cetobacterium | 0.0059 | 0.0011 | 0.0146 | 0.0016 | 0.0070 | 0.0060 |
| Ruminococcaceae_UCG-012 | 0.0135 | 0.0216 | 0.0264 | 0.0097 | 0.0162 | 0.0175 |
| Lachnoclostridium_1 | 0.0092 | 0.0049 | 0.0140 | 0.0130 | 0.0038 | 0.0090 |
| Mogibacterium | 0.0162 | 0.0086 | 0.0146 | 0.0189 | 0.0103 | 0.0137 |
| [Eubacterium]_nodatum_group | 0.0211 | 0.0162 | 0.0151 | 0.0238 | 0.0076 | 0.0167 |
| Solobacterium | 0.0157 | 0.0049 | 0.0167 | 0.0167 | 0.0130 | 0.0134 |
| Rhizobium | 0.0070 | 0.0124 | 0.0211 | 0.0027 | 0.0022 | 0.0091 |
| Oribacterium | 0.0200 | 0.0184 | 0.0227 | 0.0264 | 0.0173 | 0.0209 |
| Lachnoclostridium | 0.0022 | 0.0005 | 0.0038 | 0.0097 | 0.0022 | 0.0037 |
| Niastella | 0.0054 | 0.0016 | 0.0205 | 0.0016 | 0.0027 | 0.0064 |
| Roseburia | 0.0167 | 0.0146 | 0.0162 | 0.0173 | 0.0103 | 0.0150 |
| hoa5-07d05_gut_group | 0.0157 | 0.0194 | 0.0232 | 0.0135 | 0.0097 | 0.0163 |
| Tyzzerella_3 | 0.0173 | 0.0113 | 0.0167 | 0.0146 | 0.0103 | 0.0140 |
| Defluviitaleaceae_UCG-011 | 0.0086 | 0.0108 | 0.0200 | 0.0086 | 0.0086 | 0.0113 |
| [Ruminococcus]_gauvreauii_group | 0.0086 | 0.0065 | 0.0146 | 0.0130 | 0.0092 | 0.0104 |
| Lachnospiraceae_FCS020_group | 0.0151 | 0.0151 | 0.0124 | 0.0146 | 0.0086 | 0.0132 |
| Coprococcus_2 | 0.0092 | 0.0081 | 0.0124 | 0.0049 | 0.0140 | 0.0097 |
| [Eubacterium]_ventriosum_group | 0.0200 | 0.0097 | 0.0130 | 0.0119 | 0.0076 | 0.0124 |
| Coprococcus_1 | 0.0097 | 0.0097 | 0.0097 | 0.0086 | 0.0049 | 0.0085 |
| Family_XIII_AD3011_group | 0.0130 | 0.0059 | 0.0146 | 0.0119 | 0.0103 | 0.0111 |
| p-1088-a5_gut_group | 0.0086 | 0.0049 | 0.0065 | 0.0038 | 0.0022 | 0.0052 |
| Erysipelotrichaceae_UCG-009 | 0.0140 | 0.0086 | 0.0157 | 0.0065 | 0.0070 | 0.0104 |
| Prevotella_7 | 0.0011 | 0.0032 | 0.0070 | 0.0113 | 0.0070 | 0.0059 |
| Anaerotruncus | 0.0086 | 0.0081 | 0.0097 | 0.0092 | 0.0054 | 0.0082 |
| Lachnospiraceae_UCG-002 | 0.0130 | 0.0070 | 0.0103 | 0.0113 | 0.0130 | 0.0109 |
| Ruminococcaceae_V9D2013_group | 0.0103 | 0.0038 | 0.0097 | 0.0076 | 0.0049 | 0.0072 |
| Atopobium | 0.0016 | 0.0027 | 0.0065 | 0.0016 | 0.0032 | 0.0031 |
| Ruminiclostridium_6 | 0.0081 | 0.0097 | 0.0178 | 0.0103 | 0.0022 | 0.0096 |
| Marvinbryantia | 0.0043 | 0.0065 | 0.0097 | 0.0076 | 0.0054 | 0.0067 |
| Desulfobulbus | 0.0049 | 0.0038 | 0.0065 | 0.0054 | 0.0032 | 0.0048 |
| Phocaeicola | 0.0000 | 0.0011 | 0.0054 | 0.0027 | 0.0081 | 0.0035 |
| Candidatus_Captivus | 0.0108 | 0.0070 | 0.0065 | 0.0032 | 0.0059 | 0.0067 |
| Zoogloea | 0.0016 | 0.0059 | 0.0065 | 0.0005 | 0.0027 | 0.0035 |
| unidentified_GR-WP33-58 | 0.0027 | 0.0032 | 0.0049 | 0.0038 | 0.0027 | 0.0035 |
| Halomonas | 0.0011 | 0.0016 | 0.0043 | 0.0005 | 0.0043 | 0.0024 |
| Romboutsia | 0.0022 | 0.0016 | 0.0016 | 0.0011 | 0.0005 | 0.0014 |
| Asteroleplasma | 0.0027 | 0.0065 | 0.0092 | 0.0054 | 0.0038 | 0.0055 |
| Lachnospiraceae_UCG-010 | 0.0070 | 0.0054 | 0.0070 | 0.0059 | 0.0011 | 0.0053 |
| Blvii28_wastewater-sludge_group | 0.0081 | 0.0076 | 0.0049 | 0.0065 | 0.0059 | 0.0066 |
| Candidatus_Soleaferrea | 0.0038 | 0.0016 | 0.0027 | 0.0043 | 0.0038 | 0.0032 |
| Tyzzerella | 0.0043 | 0.0032 | 0.0022 | 0.0032 | 0.0027 | 0.0031 |
| Streptococcus | 0.0054 | 0.0022 | 0.0027 | 0.0038 | 0.0000 | 0.0028 |
| Kandleria | 0.0022 | 0.0005 | 0.0011 | 0.0043 | 0.0005 | 0.0017 |
| unidentified_Rhodospirillaceae | 0.0005 | 0.0000 | 0.0038 | 0.0005 | 0.0005 | 0.0011 |
| Syntrophococcus | 0.0027 | 0.0038 | 0.0005 | 0.0022 | 0.0011 | 0.0021 |
| Brevundimonas | 0.0011 | 0.0022 | 0.0054 | 0.0011 | 0.0005 | 0.0021 |
| Blautia | 0.0011 | 0.0022 | 0.0005 | 0.0016 | 0.0027 | 0.0016 |
| Ruminococcaceae_UCG-004 | 0.0043 | 0.0022 | 0.0016 | 0.0022 | 0.0032 | 0.0027 |
| Turicibacter | 0.0016 | 0.0032 | 0.0005 | 0.0011 | 0.0000 | 0.0013 |
| Candidatus_Bacilloplasma | 0.0011 | 0.0032 | 0.0005 | 0.0038 | 0.0005 | 0.0018 |
| Ruminococcaceae_UCG-009 | 0.0011 | 0.0038 | 0.0022 | 0.0016 | 0.0011 | 0.0019 |
| Mannheimia | 0.0022 | 0.0005 | 0.0038 | 0.0011 | 0.0005 | 0.0016 |
| Coxiella | 0.0027 | 0.0016 | 0.0011 | 0.0011 | 0.0022 | 0.0017 |
| Alysiella | 0.0027 | 0.0005 | 0.0011 | 0.0005 | 0.0022 | 0.0014 |
| Moraxella | 0.0016 | 0.0027 | 0.0005 | 0.0011 | 0.0005 | 0.0013 |
| Anaerofustis | 0.0022 | 0.0032 | 0.0022 | 0.0027 | 0.0011 | 0.0023 |
| Others | 39.2039 | 38.7009 | 34.8226 | 27.4147 | 31.0361 | 34.2356 |

The treatments HS1, HS2, HS3, HS4 and HS5 refer to the C:F ratios of 0:100, 15:85, 30:70, 45:55 and 60:40, respectively.

**Table S5** The relative abundance of the 8 most dominant bacteria at the phylum level

| Item | Groups (%)^b^ | | | | | SEM | P-values ^c^ |
| --- | --- | --- | --- | --- | --- | --- | --- |
|  | HS1 | HS2 | HS3 | HS4 | HS5 |  |  |
| *Bacteroidetes* | 51.28^bc^ | 44.88^c^ | 48.29^c^ | 67.50^a^ | 63.17^ab^ | 6.74 | <0.05 |
| *Proteobacteria* | 20.24^ab^ | 26.48^a^ | 20.83^ab^ | 10.90^b^ | 15.16^b^ | 6.93 | <0.05 |
| *Firmicutes* | 14.01 ^a^ | 12.04 ^a^ | 16.25^a^ | 14.75 ^a^ | 10.37 ^a^ | 3.68 | >0.05 |
| *Lentisphaerae* | 5.22 ^a^ | 6.69 ^a^ | 4.31^a^ | 3.38 ^a^ | 7.15 ^a^ | 1.87 | >0.05 |
| *Fibrobacteres* | 0.98 ^a^ | 1.74 ^a^ | 0.89 ^a^ | 0.56 ^a^ | 0.87 ^a^ | 0.36 | >0.05 |
| *Cyanobacteria* | 0.90^a^ | 1.01 ^a^ | 1.19 ^a^ | 0.75 ^a^ | 1.59 ^a^ | 0.21 | >0.05 |
| *Acidobacteria* | 1.79^a^ | 1.48^a^ | 1.70^a^ | 0.0098^b^ | 0.0032^b^ | 0.11 | <0.05 |
| *Actinobacteria* | 1.15^b^ | 1.34^b^ | 2.13^a^ | 0.03^c^ | 0.018^c^ | 0.12 | <0.05 |

^a^ letters which differ in the same row indicate a significant difference between values within a row.

^b^ Groups HS1, HS2, HS3, HS4 and HS5 refer to the C:F ratios of 0:100, 15:85, 30:70, 45:55 and 60:40, respectively.

^c^ Values in the same row with different superscripts are significantly different (P<0.05)

**Table S6** The effect of increases dietary concentrate on the rumen bacteria at the genus level

| Item | | Groups (%)^a^ | | | | | | SEM | P-values ^b^ |
| --- | --- | --- | --- | --- | --- | --- | --- | --- | --- |
|  |  | HS1 | | HS2 | HS3 | HS4 | HS5 |  |  |
| | *Prevotella_1* | | --- | | | | 19.62^b^ | 18.31^b^ | 21.80^ab^ | 39.17^a^ | 35.15^a^ | 3.10 | <0.05 |
| *Succinivibrionaceae_UCG-002* | | | 5.29^b^ | 11.02^a^ | 6.05^ab^ | 6.12^ab^ | 7.08^ab^ | 2.25 | >0.05 |
| *Ruminobacter* | 7.59^a^ | | | 6.09^b^ | 7.68^ab^ | 3.44^ab^ | 5.36^ab^ | 1.13 | >0.05 |
| *Rikenellaceae_RC9_gut_group* | 6.21^a^ | | | 4.68^a^ | 5.41^a^ | 3.51^a^ | 3.85^a^ | 1.23 | >0.05 |
| *Erysipelotrichaceae_UCG-004* | 1.53^a^ | | | 1.15^a^ | 1.21^a^ | 0.88^a^ | 0.68^a^ | 0.41 | >0.05 |
| *Prevotellaceae_UCG-003* | 2.65^a^ | | | 1.97^a^ | 2.19^a^ | 3.21^a^ | 3.22^a^ | 0.25 | >0.05 |
| *Succinivibrio* | 0.80^b^ | | | 1.69^a^ | 0.82^b^ | 0.36^b^ | 1.11^ab^ | 0.06 | >0.05 |
| *Fibrobacter* | 0.98^a^ | | | 1.74^a^ | 0.89^a^ | 0.56^a^ | 0.87^a^ | 0.24 | >0.05 |
| *Prevotellaceae_UCG-001* | 0.96^a^ | | | 0.76^a^ | 1.10^a^ | 1.70^a^ | 1.63^a^ | 0.25 | >0.05 |
| *Ruminococcus_2* | 0.54^a^ | | | 0.38^a^ | 0.59^a^ | 1.26^a^ | 0.41^a^ | 0.14 | >0.05 |
| *Christtensenellaceae_UCG-001* | 1.18^a^ | | | 0.96^a^ | 1.60^a^ | 1.34^a^ | 1.02^a^ | 0.50 | >0.05 |
| *Ruminococcaceae_NK4A214_group* | 1.05^a^ | | | 0.88^a^ | 1.49^a^ | 1.24^a^ | 0.80^a^ | 0.41 | >0.05 |

^a^ Groups HS1, HS2, HS3, HS4 and HS5 refer to the C:F ratios of 0:100, 15:85, 30:70, 45:55 and 60:40, respectively.

^b^ Values in the same row with different superscripts are significantly different (P<0.05).
